# Managing Drug Interactions in Cancer Therapy: A Guide for the Advanced Practitioner

**Published:** 2017-09-01

**Authors:** Christopher J. Campen, Wendy H. Vogel, Pooja J. Shah

**Affiliations:** 1 Greenville Health System Cancer Institute—Pharmacy, Greenville, South Carolina;; 2 Wellmont Cancer Institute, Kingsport, Tennessee;; 3 Department of Pharmacy, Wake Forest Baptist Health Medical Center, Winston-Salem, North Carolina

## Abstract

Mrs. P is a 30-year-old woman who presented to our bone marrow transplant program with myelodysplastic syndrome (MDS). She received a haploidentical allogeneic stem cell transplant with a conditioning regimen consisting of busulfan and cyclophosphamide. This treatment was followed by post-transplant immunosuppression for graft-versus-host disease (GVHD) with cyclophosphamide, mycophenolate mofetil (MMF), and tacrolimus (see Table 1 for medication list). Tacrolimus levels were monitored twice a week with adjustment to a goal range of between 5 and 10 ng/mL. We initiated tacrolimus at a dose of 0.03 mg/kg by mouth twice daily (rounded to 2 mg by mouth twice daily). Drug interactions were assessed by the clinical pharmacist prior to admission, routinely with medication changes, and then upon discharge.

Drug-interaction related risk factors include the use of drugs that are significantly impacted by inhibition or induction of drug metabolism (tyrosine kinase inhibitors [TKIs]), the use of drugs that have a significant inhibitory or inducing capacity of drug metabolism (certain antifungal medications), and the use of drugs with a narrow therapeutic window such as warfarin. Patient-specific risk factors include older age, renal or hepatic dysfunction, hematologic cancers, and the use of many prescribed medications (Panesar, 2011).

One retrospective drug review in cancer patients showed a high frequency of drug interactions. A total of 278 patients were reviewed, of which 40% of patients had reported potential drug interactions with their chemotherapy (van Leeuwen et al., 2011). Although this shows a high risk of drug interactions in cancer patients, it is unknown from this study what percentage of interactions were clinically relevant. In this article, we will introduce concepts and use clinically relevant examples to highlight the risk of drug interactions in patients with cancer.

Drug-drug interactions are common, not only in the oncology setting but also in the older adult population, and may be responsible for up to 4% of deaths in hospitalized oncology patients (Buajordet, Ebbesen, Erikssen, Brors, & Hilberg, 2001). A study by Van Leeuwen and associates (2013) noted that more than half of ambulatory patients with cancer had at least one potential drug interaction. One-third of ambulatory patients with cancer had a major potential drug interaction that could result in serious clinical consequences.

**Table 1:**
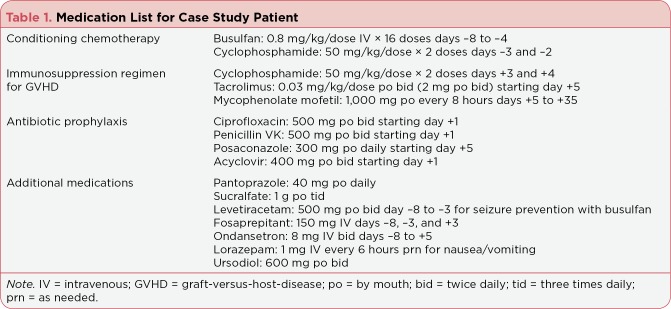
Medication List for Case Study Patient

**Table 2:**
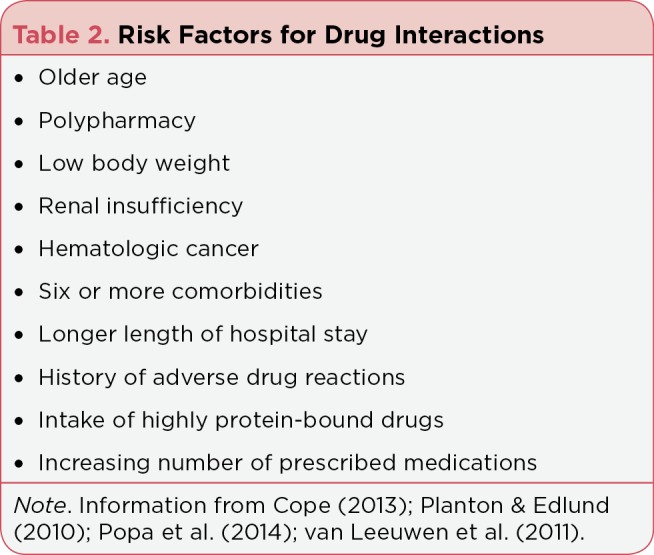
Risk Factors for Drug Interactions

Identified risk factors for drug interactions are listed in Table 2. One universal identified risk factor is an age-related change, including changes in the gastrointestinal tract (increased or decreased absorption), decreases in body fat that may influence the length of time a drug remains in the body, and decreased hepatic and renal function. A study by Popa and colleagues (2014) examined records of 244 patients who were 70+ years of age and undergoing chemotherapy. This study found 75% of patients receiving chemotherapy had a potential for a serious drug interaction involving chemotherapeutic agents.

Other risk factors include polypharmacy, defined as the use of more medications than often medically required, and the increasing number of doses of a medication per day (Cope, 2013; Planton & Edlund, 2010; Popa et al., 2014; van Leeuwen et al., 2011). As many as 80% of oncology patients utilize over-the-counter medications (Van Leeuwen et al., 2011), and these agents are not often recorded in the patient’s medical record. Patients with cancer are at a higher risk due to the increasing number of daily medications—both oncologic drug(s) as well as supportive medications.

Many new agents approved for the treatment of cancer are orally administered, indicating they are under the influence of pharmacokinetic drug interactions including absorption, distribution, metabolism, and excretion (ADME), which can reduce their effectiveness or increase toxicity. In fact, 60% of new agents approved for cancer treatment by the US Food and Drug Administration (FDA) between 2012 and 2014 were orally administered (FDA, 2017a). Most of these drugs are significantly impacted by pharmacokinetic drug interactions.

In this article, we will focus on pharmacokinetic drug interactions, but it is important to understand that other types of drug interactions such as pharmacodynamic interactions may occur. Simply stated, a pharmacokinetic interaction is the effect of the body on the drug, and a pharmacodynamic interaction is the drug’s effect on the body (Beijnen & Schellens, 2004). Pharmacodynamic drug interactions are actually very common, and such examples include the use of multiple central nervous systems (CNS) depressants or the combination of nonsteroidal anti-inflammatory drugs (NSAIDs) and angiotensin-converting enzyme (ACE) inhibitors. 

## PHARMACOKINETIC DRUG INTERACTIONS

**Absorption**

The absorption of various oral chemotherapy agents is often influenced by multiple factors such as food and acid-suppressive agents. Ultimately, these factors can impact the solubility and bioavailability of chemotherapy agents (Halfdanarson & Jatoi, 2010). For example, many oral TKIs are influenced by gastric pH changes, as seen in Tables Table 3 and Table 4. Specifically, Table Table 4 illustrates how the pH-dependent solubility of dasatinib (Sprycel) decreases as pH increases (Bristol-Myers Squibb, 2008; Eley et al., 2009).

**Table 3:**
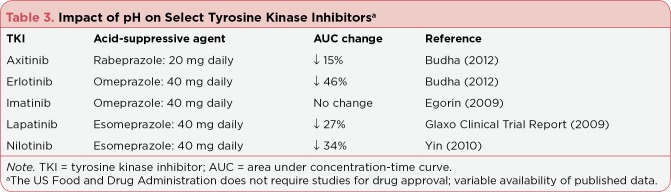
Impact of pH on Select Tyrosine Kinase Inhibitors^a^

**Table 4:**
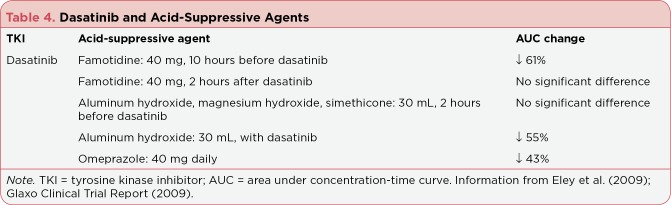
Dasatinib and Acid-Suppressive Agents

There are ways to mitigate the impact of acid suppression on drug absorption. They include the use of H2 blockers at specific times around administration of the TKI, as described in Table 3. Another reported option is to use a beverage that decreases the stomach pH for a short period such as a cola beverage (van Leeuwen et al., 2016). Furthermore, food can significantly alter the absorption of chemotherapy agents; however, the effect of food is not consistent among all chemotherapy agents, as illustrated in Table 5 (Koch et al., 2009; Reigner et al., 1998). 

**Table 5:**
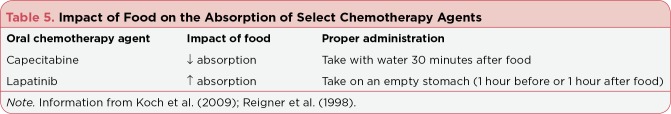
Impact of Food on the Absorption of Select Chemotherapy Agents

**Distribution**

Specific drug characteristics such as high protein binding (> 90%), narrow therapeutic index, high hepatic extraction ratio, and intravenous dosage forms may increase the likelihood of altered distribution. In particular, the impact of plasma protein binding is often overemphasized in the literature and training (Rolan, 1994). For example, TKIs are highly protein bound, but there is minimal evidence of major interactions with drugs that have the ability to displace them from the protein-binding sites.

**Metabolism**

Metabolism primarily occurs in the liver involving cytochrome P450 enzymes. Multiple drugs (refer to Table 6) competitively inhibit or induce cytochrome P450 enzyme–binding sites. This can alter the metabolism of oral and intravenous chemotherapy agents, ultimately influencing their efficacy and safety (Guengerich, 2008; Zanger & Schwab, 2013). 

**Table 6:**
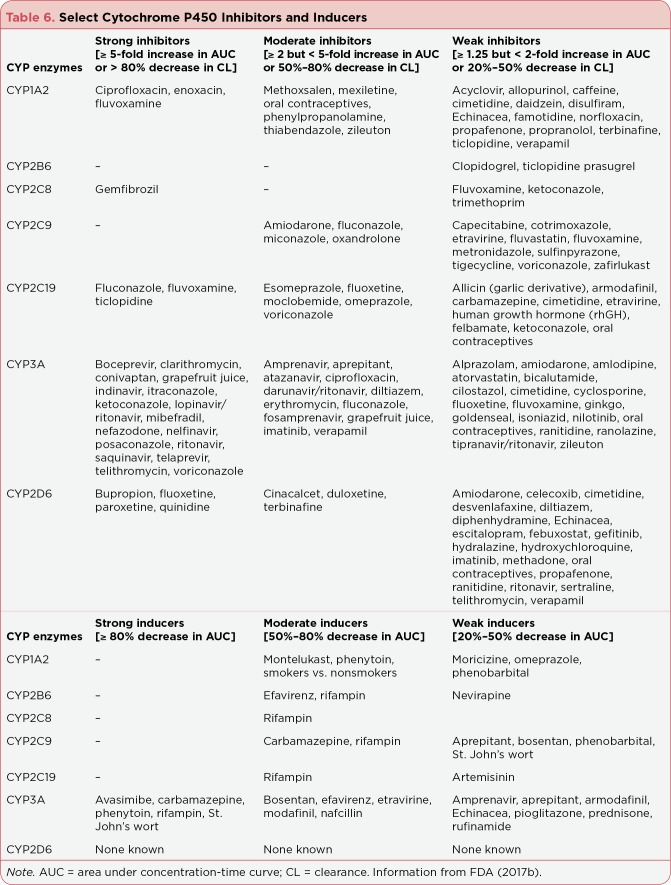
Select Cytochrome P450 Inhibitors and Inducers

For example, as illustrated in Table 6, antifungals such as voriconazole, posaconazole, and ketoconazole are very strong cytochrome P450 3A4 inhibitors, which interact with a large majority of TKIs. Certain TKIs such as ibrutinib (Imbruvica) and everolimus (Afinitor) could have profound toxicity if administered with strong inhibitors of CYP 3A4 (Kovarik et al., 2005; de Jong et al., 2015). On the other hand, rifampin or other strong inducers of CYP3A4 could significantly decrease the activity of many of the TKIs, as shown in Table 7. For these reasons, we recommend careful assessment for drug interactions any time a patient starts treatment with TKIs.

**Table 7:**
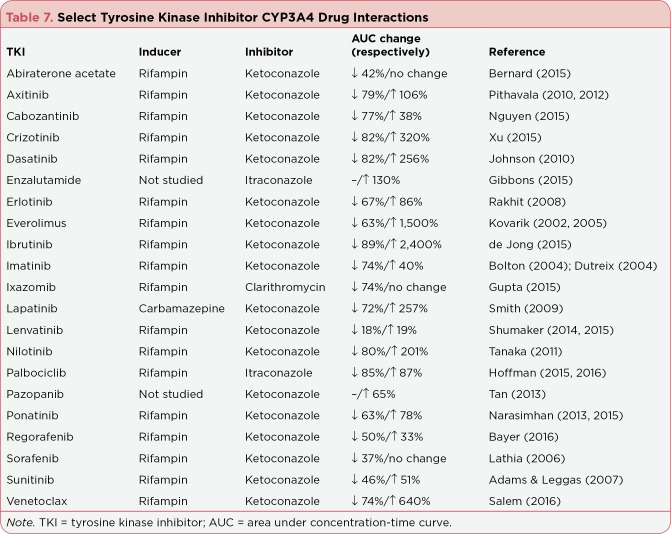
Select Tyrosine Kinase Inhibitor CYP3A4 Drug Interactions

**Elimination**

Elimination occurs mainly via the kidneys or bile. A small portion of chemotherapy agents such as methotrexate and cisplatin are primarily removed via elimination from the kidneys. High-dose methotrexate treatment can cause severe harm and even death in patients who have difficulty eliminating methotrexate and/or active metabolites of methotrexate. Certain drugs such as specific antibacterials, proton pump inhibitors, and NSAIDs can reduce the elimination of methotrexate (Ferreri et al., 2004; Fjeldborg, Sorensen, & Helkjaer, 1986; Hammor & Hasan, 2013).

## PREVENTION AND MANAGEMENT STRATEGIES FOR ADVANCED PRACTITIONERS

All oncology advanced practitioners (AP) have a vital role in the prevention, early detection, and prompt management of drug-drug interactions. As the number of oral oncologic agents increases, more safety issues and adherence issues will abound. The first step in this process of prevention and early detection of any adverse drug reaction is a full medication and health history review. The patient is instructed to bring any medication—prescribed or over-the-counter—to his clinic visit. The drug names, dosages, and schedule are documented. Any herbal supplements and/or vitamins should be documented along with dosages. Specific vernacular may be utilized to address sociocultural diversities (e.g., words such as "natural" products, folk medicine, or "home remedies"; salves; creams; and potions; Ben-Arye, Halabi, Attias, Goldstein, & Schiff, 2014).

Medical records from other health-care providers should also be examined, including clinic notes, hospitalization records, and emergency department (ED) reports. It is also important to note any potential drug absorption issues due to previous surgeries, feeding tubes, or diseases such as Crohn’s disease. 

It is estimated that up to 90% of patients use some sort of alternative or complementary medicines or therapies (Arslan, Tural, & Akar, 2013; Mao, Palmer, Healy, Desai, & Amsterdam, 2011; Naing et al., 2011; Yates et al., 2005). Yet the majority of these patients do not disclose this information to their health-care providers (Mao et al., 2011; Oh et al., 2010; Yates et al., 2005; Yildirim, 2010).

The FDA ensures the safety and efficacy of a drug released to the public. Nutritional products and supplements are exempt from this review process, however (Vogel, 2011). Patients do not understand this concept and often assume the lack of FDA regulation makes these products "safe." Unfortunately, ingredients in the products can be variable and unspecified, and there can be a lack of quality control—meaning there can be meaningful differences in the amounts of the product between different batches (Arslan et al., 2013). The AP must ensure patients understand the importance of disclosing any and all alternative/complementary therapies. 

There are risk assessment tools to assist APs to prevent, monitor for, and/or allow early identification of symptoms (Table 8). These tools may prompt APs to prescribe an appropriate medication or prevent the prescription of an inappropriate prescription. Other tools can aid APs in evaluating a medication’s potential effect on a patient’s functional and disease status. Cope (2013) noted 10 essential assessment elements to evaluate medications in older adults (Table 9). 

**Table 8:**
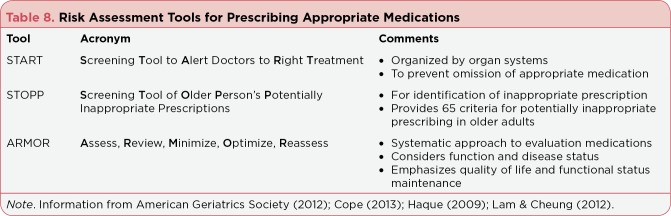
Risk Assessment Tools for Prescribing Appropriate Medications

**Table 9:**
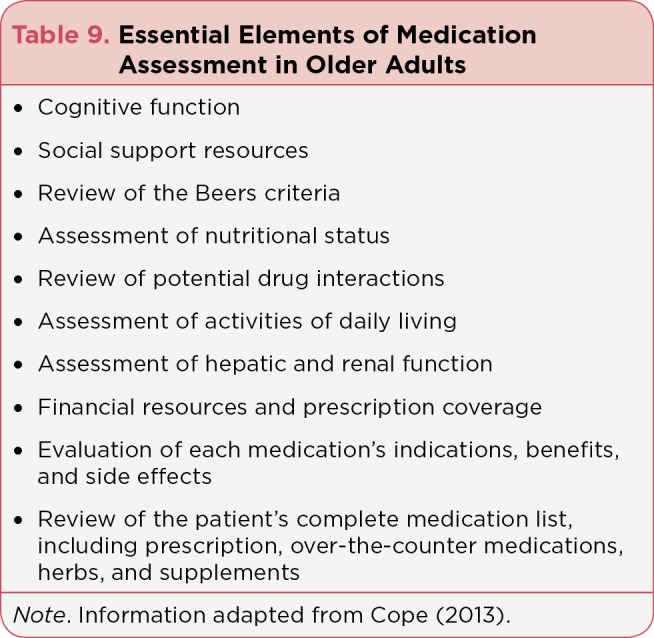
Essential Elements of Medication Assessment in Older Adults

The AP risk assessment not only includes a thorough medication review, but also the documentation of any side effects experienced by a patient. Patients should be questioned about any previous adverse events from any therapy. Assessment for substance abuse is also important, as drug metabolism may be affected. For example, smoking can induce drug-metabolizing enzymes of cytochrome P450, thus decreasing the efficacy of some oncologic agents as well as other categories of drugs (O’Malley, King, Conte, Ellingrod, & Ramnath, 2014; Sohn et al., 2015). Subcutaneous medications may have suboptimal absorption due to smoking effects. The stimulatory effects of smoking could lessen the effects of drugs such as the benzodiazepines and opioids. If a patient should suddenly quit smoking, the practitioner must maintain alertness to the possibility of a drug overdose due to increased drug exposure, such as with methadone (Zevin & Benowitz, 1999).

Thorough patient education, including proper dosing and scheduling instructions, is imperative to decrease potential drug interactions. Written information as well as verbal instructions are needed. Reminder devices such as a smartphone or an alarm clock could be helpful. Pill boxes may be useful, but many oral oncologic agents should not be placed in pill boxes, but left in the original container protected from light and moisture (Drug Information Service, University of Utah Hospitals and Clinics, 2016). The prescribing information may be consulted for details about medication storage and handling. 

Ideally, a drug-drug interaction is prevented. Up to 30% of adverse drug events in the outpatient setting are preventable (Gurwitz et al., 2003). Any prescription is carefully evaluated. Limiting the number of medications in older adults can reduce the risk of drug-drug interaction. Six or more medications increases the risk of an adverse drug event times four (Pretorius, Gataric, Swedlund, and Miller, 2013). Each new medication prescribed adds more than one adverse drug event a year. Multiple prescribers also increase adverse drug events; each additional prescriber increases the risk of an adverse drug event by 30% (Pretorius et al., 2013).

Every prescriber should share records and medication lists. Prescribers should avoid treating every side effect with another medication, considering if the dose of the offending medication could be decreased or changed to another medication (Pretorius et al., 2013). When a new medication is prescribed, a follow-up visit 2 to 4 weeks after initiating the medication is in order. In older adults, the Beers criteria should be observed (American Geriatrics Society, 2012).

The Beers criteria give a comprehensive list of medications to be avoided or used with caution in older adults. Drugs that are on this list include benzodiazepines, diphenhydramine, ibuprofen, megestrol acetate, metoclopramide, promethazine, sliding-scale insulin, and zolpidem among others. Special attention should be given to those with a history of an adverse drug event, those who are nonadherent, those who have cognitive impairment or psychiatric disease, those who have substance abuse problems, and those who live alone. 

Before increasing a dose of a medication due to seemingly suboptimal effect, APs must consider whether nonadherence is an issue. Any unnecessary medications should be discontinued. Recommended lab monitoring for certain medications should be followed according to the FDA prescribing information. When prescribing a medication, it is recommended to avoid starting more than one medication at a time (Pretorius et al., 2013). 

Although prescribers and patients must be knowledgeable about potential drug adverse reactions, the office staff must also be educated about oral oncologic agents and drug-drug interactions. Telephone triage personnel must be educated about the signs of a potential drug interaction and promptly intervene if one is suspected. The medical oncology office staff should have an oral medication adherence assessment protocol and dedicated nursing staff for oral regimens (Moody & Jackowski, 2010). Clinical decision support systems may improve prescribing quality as well by alerting the prescriber to potential drug interactions or dosages that might be incorrect. However, APs should beware of "alert fatigue," which can occur when there is poor alert specificity (Seidling et al., 2014; Weingart, Zhu, Young-Hong, Vermilya, & Hassett, 2014). 

The oncology AP should maintain a high index of suspicion for a drug-drug interaction. Some common signs of an adverse drug event might include a fall; orthostatic hypotension; heart failure; delirium or cognitive impairment; a change in daily functioning; a hospital admission; or exaggerated common adverse events (Pretorius et al., 2013). Notation should be made of the timing of symptoms after a new medication starts or after a dose change.

If a drug interaction is noted, the AP should evaluate the clinical significance of the event. The number of drugs involved should be noted. Options for management should then be reviewed and may include removal of the offending agent, removal of the affected agent, potential dose adjustments of medication, or prescription of an alternative agent(s). 

## CASE STUDY

Patients with leukemia are at a heightened risk of drug interactions due to the frequent use of medications that interact with cytochrome P450 enzymes, such as antifungal medications. Posaconazole, for example, is a broad-spectrum azole antifungal and a strong inhibitor of the CYP3A4 isoenzyme. Multiple medications the patient in our case study received were metabolized at least partially by CYP3A4, including cyclophosphamide and tacrolimus. Posaconazole was not initiated until day +5 after stem cell transplantation, to reduce the risk of a potential drug interaction with cyclophosphamide, which was administered on days –3, –2, +3, and +4. Although posaconazole has not been studied in combination with cyclophosphamide, itraconazole has been shown to increase levels of the potentially more toxic metabolites (Marr et al., 2004).

Tacrolimus is an immunosuppressant used to decrease the risk of graft-versus-host disease (GVHD), a common complication of stem cell transplantation. Tacrolimus is metabolized predominately by CYP3A4; therefore, dosing requirements are significantly decreased (> 50%) when it is used concomitantly with strong inhibitors of CYP3A4 (El-Dahshan, Bakr, Donia, Badr, & Sobh, 2004). It is important to monitor levels meticulously, as subtherapeutic levels may increase the risk of GVHD, whereas supratherapeutic levels may increase the risk of kidney dysfunction. Table 10 includes tacrolimus levels and doses throughout the inpatient admission and in the clinic.

**Table 10:**
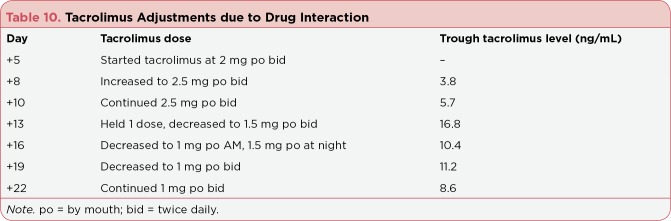
Tacrolimus Adjustments due to Drug Interaction

Tacrolimus levels initially were subtherapeutic. Over time, however, the posaconazole decreased tacrolimus metabolism through inhibition of CYP3A4. The full impact of changes in cytochrome P450 enzyme activity and a clinical interaction may not be immediately apparent, due to a delay in hepatic enzyme inhibition caused by the posaconazole and a lag in the increase in tacrolimus drug levels. This example highlights the pharmacovigilance necessary and the role APs play in monitoring patients for critical drug interactions.

## References

[1] Adams Val R, Leggas Markos (2007). Sunitinib malate for the treatment of metastatic renal cell carcinoma and gastrointestinal stromal tumors.. *Clinical therapeutics*.

[2] (2012). American Geriatrics Society updated Beers Criteria for potentially inappropriate medication use in older adults.. *Journal of the American Geriatrics Society*.

[3] Arslan Deniz, Tural Deniz, Akar Emre (2013). Herbal administration and interaction of cancer treatment.. *Journal of palliative medicine*.

[4] Bayer HealthCare Pharmaceuticals, Inc. (2016). Stivarga (regorafenib) package insert.. http://labeling.bayerhealthcare.com/html/products/pi/Stivarga_PI.pdf.

[5] Beijnen Jos H, Schellens Jan H M (2004). Drug interactions in oncology.. *The Lancet. Oncology*.

[6] Ben-Arye Eran, Halabi Inbal, Attias Samuel, Goldstein Lee, Schiff Elad (2014). Asking patients the right questions about herbal and dietary supplements: Cross cultural perspectives.. *Complementary therapies in medicine*.

[7] Bernard Apexa, Vaccaro Nicole, Acharya Milin, Jiao James, Monbaliu Johan, De Vries Ronald, Stieltjes Hans, Yu Margaret, Tran Namphuong, Chien Caly (2015). Impact on abiraterone pharmacokinetics and safety: Open-label drug-drug interaction studies with ketoconazole and rifampicin.. *Clinical pharmacology in drug development*.

[8] Bolton Ann E, Peng Bin, Hubert Martine, Krebs-Brown Axel, Capdeville Renaud, Keller Urs, Seiberling Michael (2004). Effect of rifampicin on the pharmacokinetics of imatinib mesylate (Gleevec, STI571) in healthy subjects.. *Cancer chemotherapy and pharmacology*.

[9] Bristol-Myers Squibb. (2008). The effect of omeprazole on the pharmacokinetics of dasatinib (BMS-354825) in healthy subjects.. http://ctr.bms.com/pdf/CA180249.pdf.

[10] Buajordet I, Ebbesen J, Erikssen J, Brørs O, Hilberg T (2001). Fatal adverse drug events: the paradox of drug treatment.. *Journal of internal medicine*.

[11] Budha N R, Frymoyer A, Smelick G S, Jin J Y, Yago M R, Dresser M J, Holden S N, Benet L Z, Ware J A (2012). Drug absorption interactions between oral targeted anticancer agents and PPIs: is pH-dependent solubility the Achilles heel of targeted therapy?. *Clinical pharmacology and therapeutics*.

[12] Cascorbi I (2012). Drug interactions—principles, examples and clinical consequences.. *Deutsches ÄrzteblattInternational*.

[13] Cope Diane G (2013). Polypharmacy in older adults: the role of the advanced practitioner in oncology.. *Journal of the advanced practitioner in oncology*.

[14] de Jong J, Skee D, Murphy J, Sukbuntherng J, Hellemans P, Smit J, Mannaert E (2015). Effect of CYP3A perpetrators on ibrutinib exposure in healthy participants.. *Pharmacology Research and Perspectives*.

[15] Drug Information Service, University of Utah Hospitals and Clinics. (2016). Oral medications that should not be packaged in the prepackage machine and oral medications that require special handling precautions.. http://www.ashp.org/DocLibrary/MemberCenter/SICP/Univ-of-Utah-do-not-prepack-list.pdf.

[16] Dutreix Catherine, Peng Bin, Mehring Guenther, Hayes Michael, Capdeville Renaud, Pokorny Rolf, Seiberling Michael (2004). Pharmacokinetic interaction between ketoconazole and imatinib mesylate (Glivec) in healthy subjects.. *Cancer chemotherapy and pharmacology*.

[17] Egorin Merrill J, Shah Dhvani D, Christner Susan M, Yerk Mara A, Komazec Kristin A, Appleman Leonard R, Redner Robert L, Miller Brian M, Beumer Jan H (2009). Effect of a proton pump inhibitor on the pharmacokinetics of imatinib.. *British journal of clinical pharmacology*.

[18] el-Dahshan Khalid Farouk, Bakr Mohamed Adel, Donia Ahmed Farouk, Badr Ali el-Sayed, Sobh Mohamed Abdel-Kader (2004). Co-administration of ketoconazole to tacrolimus-treated kidney transplant recipients: a prospective randomized study.. *Nephrology, dialysis, transplantation : official publication of the European Dialysis and Transplant Association - European Renal Association*.

[19] Eley Timothy, Luo Feng R, Agrawal Shruti, Sanil Ashish, Manning James, Li Tong, Blackwood-Chirchir Anne, Bertz Richard (2009). Phase I study of the effect of gastric acid pH modulators on the bioavailability of oral dasatinib in healthy subjects.. *Journal of clinical pharmacology*.

[20] Ferreri A J M, Guerra E, Regazzi M, Pasini F, Ambrosetti A, Pivnik A, Gubkin A, Calderoni A, Spina M, Brandes A, Ferrarese F, Rognone A, Govi S, Dell'Oro S, Locatelli M, Villa E, Reni M (2004). Area under the curve of methotrexate and creatinine clearance are outcome-determining factors in primary CNS lymphomas.. *British journal of cancer*.

[21] Fjeldborg P, Sørensen J, Helkjaer P E (1986). The long-term effect of cisplatin on renal function.. *Cancer*.

[22] Gibbons Jacqueline A, de Vries Michiel, Krauwinkel Walter, Ohtsu Yoshiaki, Noukens Jan, van der Walt Jan-Stefan, Mol Roelof, Mordenti Joyce, Ouatas Taoufik (2015). Pharmacokinetic Drug Interaction Studies with Enzalutamide.. *Clinical pharmacokinetics*.

[23] Glaxo Clinical Trial Report. (2009). An open-label, single sequence study to examine the effects of esomeprazole on the pharmacokinetics of orally administered lapatinib in subjects with metastatic ErbB2 positive breast cancer.. http://www.gsk-clinicalstudyregister.com/files2/9ed5ea0d-8365-439f-98e4-96119b2189e9.

[24] Guengerich F Peter (2008). Cytochrome p450 and chemical toxicology.. *Chemical research in toxicology*.

[25] Gupta N, Hanley M, Venkatakrishnan K, Bessudo A, Sharma S, O’Neil B, Nemunaitis J (2015). Abstract B147: A phase 1 drug-drug interaction study between ixazomib, an oral proteasome inhibitor, and rifampin in patients with advanced solid tumors.. *Molecular Cancer Therapeutics*.

[26] Gurwitz Jerry H, Field Terry S, Harrold Leslie R, Rothschild Jeffrey, Debellis Kristin, Seger Andrew C, Cadoret Cynthia, Fish Leslie S, Garber Lawrence, Kelleher Michael, Bates David W (2003). Incidence and preventability of adverse drug events among older persons in the ambulatory setting.. *JAMA*.

[27] Halfdanarson Thorvardur R, Jatoi Aminah (2010). Oral cancer chemotherapy: the critical interplay between patient education and patient safety.. *Current oncology reports*.

[28] Hammor Y A, Hasan Y (2013). Prevention and management of high-dose methotrexate toxicity. *Journal of Cancer Science and Therapy*.

[29] Haque R (2009). ARMOR: A tool to evaluate polypharmacy in elderly persons.. *Annals of Long-Term Care*.

[30] Hoffman J T, Loi C M, O’Gorman M, Plotka A, Kosa M, Jakubowska A, Wang D D (2016). Abstract LB-196: A phase I open-label, fixed-sequence, two-period crossover study of the effect of multiple doses of itraconazole on palbociclib (PD–0332991) pharmacokinetics in healthy volunteers.. *Cancer Research*.

[31] Hoffman J T, Plotka A, O’Gorman M, Chang A, Kosa M, Loi C M, Wang D D (2015). Abstract 4515: A phase 1 randomized, open-label, fixed-sequence, 2-period study of the effect of multiple doses of rifampin on palbociclib (PD-0332991) pharmacokinetics in healthy volunteers.. *Cancer Research*.

[32] Johnson Faye M, Agrawal Shruti, Burris Howard, Rosen Lee, Dhillon Navneet, Hong David, Blackwood-Chirchir Anne, Luo Feng R, Sy Oumar, Kaul Sanjeev, Chiappori Alberto A (2010). Phase 1 pharmacokinetic and drug-interaction study of dasatinib in patients with advanced solid tumors.. *Cancer*.

[33] Koch Kevin M, Reddy Nandi J, Cohen Roger B, Lewis Nancy L, Whitehead Bonnie, Mackay Kathleen, Stead Andrew, Beelen Andrew P, Lewis Lionel D (2009). Effects of food on the relative bioavailability of lapatinib in cancer patients.. *Journal of clinical oncology : official journal of the American Society of Clinical Oncology*.

[34] Kovarik J M, Beyer D, Bizot M N, Jiang Q, Shenouda M, Schmouder R L (2005). Blood concentrations of everolimus are markedly increased by ketoconazole.. *Journal of clinical pharmacology*.

[35] Kovarik John M, Hartmann Stefan, Figueiredo Joaquim, Rouilly Marisel, Port Andreas, Rordorf Christiane (2002). Effect of rifampin on apparent clearance of everolimus.. *The Annals of pharmacotherapy*.

[36] Lam May P S, Cheung Bernard M Y (2012). The use of STOPP/START criteria as a screening tool for assessing the appropriateness of medications in the elderly population.. *Expert review of clinical pharmacology*.

[37] Lathia Chetan, Lettieri John, Cihon Frank, Gallentine Martha, Radtke Martin, Sundaresan Pavur (2006). Lack of effect of ketoconazole-mediated CYP3A inhibition on sorafenib clinical pharmacokinetics.. *Cancer chemotherapy and pharmacology*.

[38] Mao Jun James, Palmer Christina Shearer, Healy Kaitlin Elizabeth, Desai Krupali, Amsterdam Jay (2011). Complementary and alternative medicine use among cancer survivors: a population-based study.. *Journal of cancer survivorship : research and practice*.

[39] Marr Kieren A, Leisenring Wendy, Crippa Fulvio, Slattery John T, Corey Lawrence, Boeckh Michael, McDonald George B (2004). Cyclophosphamide metabolism is affected by azole antifungals.. *Blood*.

[40] Moody Mendy, Jackowski Joyce (2010). Are patients on oral chemotherapy in your practice setting safe?. *Clinical journal of oncology nursing*.

[41] Naing Aung, Stephen Saneese K, Frenkel Moshe, Chandhasin Chandtip, Hong David S, Lei Xiudong, Falchook Gerald, Wheler Jennifer J, Fu Siqing, Kurzrock Razelle (2011). Prevalence of complementary medicine use in a phase 1 clinical trials program: the MD Anderson Cancer Center Experience.. *Cancer*.

[42] Narasimhan Narayana I, Dorer David J, Davis Jeffrey, Turner Christopher D, Sonnichsen Daryl (2015). Evaluation of the effect of multiple doses of rifampin on the pharmacokinetics and safety of ponatinib in healthy subjects.. *Clinical pharmacology in drug development*.

[43] Narasimhan Narayana I, Dorer David J, Niland Katie, Haluska Frank, Sonnichsen Daryl (2013). Effects of ketoconazole on the pharmacokinetics of ponatinib in healthy subjects.. *Journal of clinical pharmacology*.

[44] Nguyen Linh, Holland Jaymes, Miles Dale, Engel Caroline, Benrimoh Natacha, O'Reilly Terry, Lacy Steven (2015). Pharmacokinetic (PK) drug interaction studies of cabozantinib: Effect of CYP3A inducer rifampin and inhibitor ketoconazole on cabozantinib plasma PK and effect of cabozantinib on CYP2C8 probe substrate rosiglitazone plasma PK.. *Journal of clinical pharmacology*.

[45] O’Malley Meaghan, King Amanda N, Conte Marisa, Ellingrod Vicki L, Ramnath Nithya (2014). Effects of cigarette smoking on metabolism and effectiveness of systemic therapy for lung cancer.. *Journal of thoracic oncology : official publication of the International Association for the Study of Lung Cancer*.

[46] Oh Byeongsang, Butow Phyllis, Mullan Barbara, Clarke Stephen, Tattersall Martin, Boyer Michael, Beale Philip, Vardy Janette, Pavlakis Nick, Larke Linda (2010). Patient-doctor communication: use of complementary and alternative medicine by adult patients with cancer.. *Journal of the Society for Integrative Oncology*.

[47] Panesar K A (2011). Typical drug interactions in oncology.. *US Pharmacist*.

[48] Pithavala Yazdi K, Tong Warren, Mount Janessa, Rahavendran Sadayappan V, Garrett May, Hee Brian, Selaru Paulina, Sarapa Nenad, Klamerus Karen J (2012). Effect of ketoconazole on the pharmacokinetics of axitinib in healthy volunteers.. *Investigational new drugs*.

[49] Pithavala Y K, Tortorici M, Toh M, Garrett M, Hee B, Kuruganti U, Ni G, Klamerus K J (2010). Effect of rifampin on the pharmacokinetics of Axitinib (AG-013736) in Japanese and Caucasian healthy volunteers.. *Cancer chemotherapy and pharmacology*.

[50] Planton Jonathan, Edlund Barbara J (2010). Strategies for reducing polypharmacy in older adults.. *Journal of gerontological nursing*.

[51] Popa Mihaela A, Wallace Kristie J, Brunello Antonella, Extermann Martine, Balducci Lodovico (2014). Potential drug interactions and chemotoxicity in older patients with cancer receiving chemotherapy.. *Journal of geriatric oncology*.

[52] Pretorius Richard W, Gataric Gordana, Swedlund Steven K, Miller John R (2013). Reducing the risk of adverse drug events in older adults.. *American family physician*.

[53] Rakhit Ashok, Pantze Michael P, Fettner Scott, Jones Hannah M, Charoin Jean-Eric, Riek Myriam, Lum Bert L, Hamilton Marta (2008). The effects of CYP3A4 inhibition on erlotinib pharmacokinetics: computer-based simulation (SimCYP) predicts in vivo metabolic inhibition.. *European journal of clinical pharmacology*.

[54] Reigner B, Verweij J, Dirix L, Cassidy J, Twelves C, Allman D, Weidekamm E, Roos B, Banken L, Utoh M, Osterwalder B (1998). Effect of food on the pharmacokinetics of capecitabine and its metabolites following oral administration in cancer patients.. *Clinical cancer research : an official journal of the American Association for Cancer Research*.

[55] Rolan P E (1994). Plasma protein binding displacement interactions—Why are they still regarded as clinically important?. *British Journal of Clinical Pharmacology*.

[56] Salem Ahmed Hamed, Agarwal Suresh K, Dunbar Martin, Enschede Sari L Heitner, Humerickhouse Rod A, Wong Shekman L (2017). Pharmacokinetics of Venetoclax, a Novel BCL-2 Inhibitor, in Patients With Relapsed or Refractory Chronic Lymphocytic Leukemia or Non-Hodgkin Lymphoma.. *Journal of clinical pharmacology*.

[57] Seidling Hanna M, Klein Ulrike, Schaier Matthias, Czock David, Theile Dirk, Pruszydlo Markus G, Kaltschmidt Jens, Mikus Gerd, Haefeli Walter E (2014). What, if all alerts were specific - estimating the potential impact on drug interaction alert burden.. *International journal of medical informatics*.

[58] Shumaker Robert C, Aluri Jagadeesh, Fan Jean, Martinez Gresel, Thompson Gary A, Ren Min (2014). Effect of rifampicin on the pharmacokinetics of lenvatinib in healthy adults.. *Clinical drug investigation*.

[59] Shumaker Robert, Aluri Jagadeesh, Fan Jean, Martinez Gresel, Thompson Gary A, Ren Min (2015). Effects of Ketoconazole on the Pharmacokinetics of Lenvatinib (E7080) in Healthy Participants.. *Clinical pharmacology in drug development*.

[60] Smith Deborah A, Koch Kevin M, Arya Nikita, Bowen Carolyn J, Herendeen Jill M, Beelen Andrew (2009). Effects of ketoconazole and carbamazepine on lapatinib pharmacokinetics in healthy subjects.. *British journal of clinical pharmacology*.

[61] Sohn Hyun Soon, Kim Hyunah, Song Im-Sook, Lim Eunjeong, Kwon Mihwa, Ha Ji-Hye, Kwon Jin-Won (2015). Evidence supporting the need for considering the effects of smoking on drug disposition and effectiveness in medication practices: a systematic narrative review.. *International journal of clinical pharmacology and therapeutics*.

[62] Tan Antoinette R, Gibbon Darlene G, Stein Mark N, Lindquist Diana, Edenfield Jeffery W, Martin Julie C, Gregory Charles, Suttle A Benjamin, Tada Hiroomi, Botbyl Jeffrey, Stephenson Joseph J (2013). Effects of ketoconazole and esomeprazole on the pharmacokinetics of pazopanib in patients with solid tumors.. *Cancer chemotherapy and pharmacology*.

[63] Tanaka Chiaki, Yin Ophelia Q P, Smith Tom, Sethuraman Venkat, Grouss Karen, Galitz Lawrence, Harrell Robert, Schran Horst (2011). Effects of rifampin and ketoconazole on the pharmacokinetics of nilotinib in healthy participants.. *Journal of clinical pharmacology*.

[64] US Food and Drug Administration. (2017b). FDA drug development and drug interactions: Table of substrates, inhibitors and inducers.. http://www.fda.gov/Drugs/DevelopmentApprovalProcess/DevelopmentResources/DrugInteractionsLabeling/ucm080499.htm.

[65] US Food and Drug Administration. (2017a). Hematology/oncology (cancer) approvals and safety notifications.. http://www.fda.gov/Drugs/InformationOnDrugs/ApprovedDrugs/ucm279174.htm.

[66] van Leeuwen R W F, Brundel D H S, Neef C, van Gelder T, Mathijssen R H J, Burger D M, Jansman F G A (2013). Prevalence of potential drug-drug interactions in cancer patients treated with oral anticancer drugs.. *British journal of cancer*.

[67] van Leeuwen Roelof W F, Peric Robert, Hussaarts Koen G A M, Kienhuis Emma, IJzerman Nikki S, de Bruijn Peter, van der Leest Cor, Codrington Henk, Kloover Jeroen S, van der Holt Bronno, Aerts Joachim G, van Gelder Teun, Mathijssen Ron H J (2016). Influence of the Acidic Beverage Cola on the Absorption of Erlotinib in Patients With Non-Small-Cell Lung Cancer.. *Journal of clinical oncology : official journal of the American Society of Clinical Oncology*.

[68] van Leeuwen R W F, Swart E L, Boven E, Boom F A, Schuitenmaker M G, Hugtenburg J G (2011). Potential drug interactions in cancer therapy: a prevalence study using an advanced screening method.. *Annals of oncology : official journal of the European Society for Medical Oncology*.

[69] Vogel W (2011). Internet oncology: Cure seekers beware!. *Journal of the Advanced Practitioner in Oncology*.

[70] Weingart Saul N, Zhu Junya, Young-Hong Joanne, Vermilya Holly Barr, Hassett Michael (2014). Do drug interaction alerts between a chemotherapy order-entry system and an electronic medical record affect clinician behavior?. *Journal of oncology pharmacy practice : official publication of the International Society of Oncology Pharmacy Practitioners*.

[71] Xu Huiping, O’Gorman Melissa, Tan Weiwei, Brega Nicoletta, Bello Akintunde (2015). The effects of ketoconazole and rifampin on the single-dose pharmacokinetics of crizotinib in healthy subjects.. *European journal of clinical pharmacology*.

[72] Yates Jennifer S, Mustian Karen M, Morrow Gary R, Gillies Leslie J, Padmanaban Devi, Atkins James N, Issell Brian, Kirshner Jeffrey J, Colman Lauren K (2005). Prevalence of complementary and alternative medicine use in cancer patients during treatment.. *Supportive care in cancer : official journal of the Multinational Association of Supportive Care in Cancer*.

[73] Yildirim Y (2010). Patterns of the use of complementary and alternative medicine in women with metastatic cancer.. *Cancer Nursing*.

[74] Yin Ophelia Q P, Gallagher Neil, Fischer Deirdre, Demirhan Eren, Zhou Wei, Golor Georg, Schran Horst (2010). Effect of the proton pump inhibitor esomeprazole on the oral absorption and pharmacokinetics of nilotinib.. *Journal of clinical pharmacology*.

[75] Zanger Ulrich M, Schwab Matthias (2013). Cytochrome P450 enzymes in drug metabolism: regulation of gene expression, enzyme activities, and impact of genetic variation.. *Pharmacology & therapeutics*.

[76] Zevin S, Benowitz N L (1999). Drug interactions with tobacco smoking. An update.. *Clinical pharmacokinetics*.

